# Understanding the dynamics of compliance to smoke-free policy regulations: Exploring the perspectives of venue owners and staff in Türkiye

**DOI:** 10.18332/tid/176226

**Published:** 2024-01-17

**Authors:** Ezgi Baltacı, Aslı Çarkoğlu, Sejal Saraf, Toker Ergüder, Gül Ergör, Mutlu Hayran, Connie Hoe

**Affiliations:** 1 Heidelberg Institute of Global Health, Faculty of Medicine, Heidelberg University, Heidelberg, Germany; 2 Department of Psychology, Kadir Has University, Istanbul, Türkiye; 3 Johns Hopkins Institute for Global Tobacco Control, Johns Hopkins Bloomberg School of Public Health, Baltimore, United States; 4 Special Initiative on NCDs and Innovation (SNI), World Health Organization Country Office, Ankara, Türkiye; 5 School of Public Health, Dokuz Eylül University, Izmir, Türkiye; 6 Cancer Institute, Hacettepe University, Ankara, Türkiye

**Keywords:** smoke-free policy, facilitators, barriers, compliance, COVID-19

## Abstract

**INTRODUCTION:**

The study aims to understand the facilitators and barriers associated with enforcing and complying with Türkiye's smoke-free policy from the perspective of hospitality venue owners and employees.

**METHODS:**

A qualitative open-ended survey was conducted in Istanbul and Ankara in 2021 with 58 respondents from 3 different districts in each city from four types of venues: restaurants, traditional coffee and waterpipe houses, and European-style cafés. The open-ended survey included questions to understand the knowledge, beliefs, and attitudes of respondents about Türkiye's smoke-free policy and their perceptions of the facilitators and/or barriers to smoke-free policy implementation and changes after COVID-19. The data were analyzed using an inductive approach to identify patterns and categorize the data into themes.

**RESULTS:**

The respondents expressed that the smoke-free policy aimed to protect employees and customers from secondhand smoke (SHS), respect human health, and improve air quality. Findings suggest that the positive attitude of venue owners and staff toward the smoke-free policy serves as a facilitator. However, fear of financial impact, customers’ negative attitudes, difficulties in meeting physical requirements, and insufficient enforcement were found to be barriers to implementing the smoke-free policy. The effects of the COVID-19 pandemic were reported as an initial increase in compliance and awareness among customers and staff, but some respondents noted negative changes due to the emotional and financial effects of prolonged restrictions. These challenges have led to decreased attention on the smoke-free policy among venue owners, staff and customers. Respondents’ suggested improvements were related to building infrastructure, such as the ventilation systems and educating the public on the harmful health effects of smoking.

**CONCLUSIONS:**

Despite the general understanding of the dangers of secondhand smoke and the smoke-free policy, this study highlights the challenges in implementing smoke-free policy measures and the continued need to raise awareness about the importance of a 100% smoke-free venue. A comprehensive approach to addressing the tobacco epidemic as a multifaceted public health issue is essential.

## INTRODUCTION

The World Health Organization (WHO) Framework Convention on Tobacco Control (FCTC) provides a foundation for countries to implement effective policies and interventions to reduce the demand for tobacco and protect people from the harmful effects of smoking^1^. Specifically, Article 8 of the WHO FCTC calls for a comprehensive smoke-free policy to eliminate tobacco smoking in all indoor public places and workplaces^2^.

Türkiye ratified the WHO FCTC in 2004 and passed a law banning smoking in all indoor public places, including bars, cafes, and restaurants, in 2008^3^. By 2013, Türkiye became the first country recognized by the WHO to implement all MPOWER (Monitor-Protect-Offer-Warn-Enforce-Raise) policies at the highest level^3^.

While enacting the policy is significant, it is also critical to implement and enforce smoke-free policies to ensure complete protection from secondhand smoke exposure. Previous compliance studies in Türkiye demonstrated low smoke-free policy compliance in coffee houses and bars/nightclubs, and recorded unhealthy levels of fine particles in the air due to smoking in these hospitality venues^4,5^. Also, only a small proportion of hospitality venue owners and employees expressed a willingness to enforce the law if they saw a violation^6^.

In early 2020, the coronavirus disease 2019 (COVID-19) pandemic brought changes^7^. All venues were closed during the first stage of the pandemic. Additionally, the WHO reported in June 2020 that smoking is associated with increased disease severity and death in hospitalized COVID-19 patients^8^, which gives merit to evaluate the implications of alterations in compliance with the smoke-free policy in hospitality venues after COVID-19 restrictions that have not been explored in the literature.

Tobacco control efforts seem to have faltered in Türkiye; recent reports of an increase in nationwide smoking rates^9^ led to concerns of non-compliance, particularly in private business, cafes, and restaurants, which had difficulties adapting^5,6^.

This study aims to understand the facilitators and barriers associated with enforcing and complying with the smoke-free policy. The research questions are: 1) ‘What are hospitality venue owners’ and employees’ knowledge, beliefs, and attitudes about Türkiye’s smoke-free policy?’; 2) ‘What do they perceive as the facilitators or barriers to smoke-free policy implementation?’; and 3) ‘How did compliance and implementation change with COVID-19?’.

## METHODS

### Study design

In this qualitative study, the data collection took place in two of the largest cities of Türkiye – Istanbul and Ankara – between July and August 2021. First, districts were selected within each city as areas of the hospitality venues: Ankara – Altındağ, Çankaya, and Tunalı; İstanbul – Güngören, Üsküdar, and Zeytinburnu. Subsequently, two points were randomly chosen within each district as the start of a walking protocol, which was used to sample the venues nearest to these points^10^. Four types of venues were observed in each area: restaurants, traditional coffee houses, European-style cafés, and waterpipe coffee houses (Table 1). The collaboration for data collection involved Johns Hopkins University, IGTC, and Turkish academic experts from Kadir Has University, Hacettepe University, and Dokuz Eylul University, in partnership with the local Omega Contract Research Organization.

**Table 1:** Participants numbers and IDs by provinces, districts, and venue types in understanding the dynamics of venue (non-)compliance with the smoke-free law in Türkiye study, 2021 (N=58)

*Province District*	*Restaurants*	*Waterpipe and traditional coffee houses*	*European style cafes*	*Total*
Istanbul Güngören	Venue owner ID: 16, 49 Staff ID: 17, 50	Venue owner ID: 46	Venue owner ID: 19 Staff ID: 18	7
Istanbul Zeytinburnu	Venue owner ID: 14, 21, 26, 56 Staff ID: 13, 20, 27, 55		Venue owner ID: 4 Staff ID: 3	10
Istanbul Uskudar	Venue owner ID: 2, 54 Staff ID: 1, 45, 53	Venue owner ID: 33 Staff ID: 32	Venue owner ID: 12, 23, 29 Staff ID: 11, 22, 28	13
Ankara Tunalı	Venue owner ID: 40, 43 Staff ID: 39, 44		Venue owner ID: 25 Staff ID: 24	6
Ankara Cankaya	Venue owner ID: 38, 42, 48 Staff ID: 8, 36, 37, 41, 47		Venue owner ID: 6, 10, 31, 34, 58 Staff ID: 5, 9, 15, 30, 35, 57	19
Ankara Altındag	Venue owner ID: 7		Venue owner ID: 51 Staff ID: 52	3
Total	30	2	25	58

A total of 58 open-ended surveys were conducted with venue owners and staff. The interview guide was designed to address key topics related to compliance, including awareness of the smoke-free law, enforcement practices, perceptions of the law, and challenges faced in adhering to the regulations^10^ (Supplementary file). This study was planned before the pandemic and was postponed during the pandemic. During this pause, we decided to add a new component to understand the effects of this global crisis on the smoke-free policy in these most vulnerable types of hospitality venues. Consequently, the questionnaire included inquiries about any modifications in compliance levels with COVID-19 regulations.

Trained interviewers conducted interviews with Turkish venue representatives, which included owners, managers, and staff. Oral consent was obtained from each key informant before the interview commenced. In cases where interviewees opted out of audio recording, detailed written notes were taken to document their responses. Nineteen interviews were audio-recorded using recording devices, and the average interview duration was 15.19 minutes. Subsequently, these audio recordings were transcribed verbatim in Turkish and then translated into English.

This study was deemed non-human research by the Johns Hopkins Bloomberg School of Public Health Institutional Review Board. It also received ethics approval from Kadir Has University in Türkiye.

### Data analysis

The data were analyzed using an inductive approach to allow for themes to emerge. The recordings and notes in Turkish were the primary sources of analysis. After becoming familiar with the data by reading the interview transcripts and listening to the audio recordings, the interview transcripts were entered into NVivo 12 software^11^. The initial coding phase began with line-by-line coding of the text, followed by focused coding to identify patterns and to categorize the data into themes^12^.

## RESULTS

We present the findings (Table 2) organized according to the following topics: 1) understanding the attitude of venue owners and staff towards the smoke-free policy and its implementation, 2) perceptions about what is working and what parts are failing in implementation, 3) what COVID-19 restrictions have changed, and 4) suggestions for improvements on implementation.

**Table 2:** The key findings with related topics in understanding the dynamics of venue (non-) compliance with the smoke-free law in Türkiye

*Topic*	*Key findings*
**Venue owner and staff understanding of the smoke-free policy and its implementation**	Respondents generally welcomed the smoke-free policy, with many acknowledging its health benefits for employees, customers, and children.
	Respondents perceived the policy to have potential, but its implementation was found to be inadequate.
	Those who believed the policy was ineffective pointed to the high number of smokers and a lack of enforcement as key challenges.
**Perceived facilitator to implementation of the smoke-free policy**	Venue owners and staff demonstrated a positive attitude and support for implementing the smoke-free policy through staff-to-customer interactions.
**Perceived barriers to implementation of the smoke-free policy**	Negative customer attitudes and the need to maintain customer satisfaction; customers may leave venues or insist on smoking indoors, even after being warned.
	Economic concerns, including potential customer loss, the costs of providing outdoor smoking spaces, and the expenses associated with heating outdoor spaces in cold weather.
	Physical properties of venues presented challenges for some venues, particularly small businesses in terms of providing smoking areas.
	Insufficient enforcement; lack of uniform and effective enforcement leads to unfair competition and perceptions of limited consequences for violations.
**COVID-19 pandemic and changes in compliance and implementation**	Venue staff and customers reported an initial increase in compliance during the early stages of the pandemic.
	The pandemic’s prolonged restrictions led to increased stress among customers, making them more likely to smoke.
	Venues faced reduced seating capacity due to social distancing regulations and were desperate for customers. As a result, some venues became less strict in enforcing the smoking ban to attract customers.
**Suggestions for better implementation**	Softening the indoor smoking ban and changing the definition of enclosed areas in the law to ease compliance such as ‘smoking areas’ or filtered air ventilation to allow smoking indoors.
	Increasing fines and enhancing regulation visit-routines for better deterrence and effectiveness, including raising fines and conducting more frequent checks by municipal police.

### Venue owner and staff understanding of the smoke-free policy and its implementation

We asked the respondents what the positives of the smoke-free policy were, to understand their attitudes. The vast majority pointed towards protecting employees from SHS and eliminating SHS for customers and children. Some participants mentioned non-smoking customers’ satisfaction with the smoke-free policy:

*‘I think these regulations work, people who smoke unintentionally violate the rights of people who do not smoke. These regulations eliminate this.’* (ID 6, Café owner, Ankara)

Some respondents believed the smoke-free policy worked, yet many saw issues with the implementation of the law. Even those who stated that the smoke-free policy was effective, mentioned that people only followed the rules and complied with the law when warned:

*‘I think it has worked. Everybody is following the rules; they do not smoke when we say smoking is prohibited here.’* (ID 15, Café staff, Ankara)

However, others highlighted the importance of having proper enforcement and the business owner’s supportive attitude for the policy to work. The respondents who thought the smoke-free policy did not work, explained that the number of smokers does not seem to have decreased, and the main barrier to the implementation of the smoke-free policy is the high percentage of smokers:

*‘I would allow smoking. No, as I said earlier, 90% of our customers smoke. Whatever they do, people still smoke. They put pictures of people with lung cancer and cerebral bleeding on it; people still smoke. I put it upside down on the table not to see them.’* (ID 50, Restaurant staff, Istanbul)

Some respondents added that the smoke-free policy did not work due to a lack of enforcement, and they thought the policy failed to prevent SHS:

*‘I think it does not work, because there is no control for implementation.’* (ID 5, Café staff, Ankara)

### Perceptions about what is working and what is not

The only part of the implementation process that is reported to be working were staff-to-customer warnings and negotiations, which were closely tied to the personal, smoke-free, supportive attitudes of the venue owners and staff. All other factors mentioned in the interviews can be considered as parts of the implementation that are failing and in need of improvement.

*Attitudes of venue owners and staff*

Participants reported that even though customers ask for ashtrays or indoor smoking, they understand and do not insist or object when the venue owners and staff say that smoking is prohibited indoors:

*‘I mean people only ask if they can smoke here. Especially at these tables by the window, when we say no, they understand we don’t have any problems.’* (ID 11, Café staff, Istanbul)

The positive attitude of venue owners/staff was mostly seen to be rooted in this type of direct intervention. Their knowledge of the harmful health effects of SHS and the perception of the smoke-free policy motivated them to intervene and helped protect both customers and employees from SHS:

*‘I think it is good in terms of protecting the health of both employees and non-smokers. I think it works to the extent that the controls are done. It can be ignored if compulsory controls are not done.’* (ID 53, Restaurant staff, Istanbul)

*Attitudes of customers*

Most of the respondents mentioned the negative attitude of customers towards the smoking ban as a barrier to the implementation of the smoke-free policy. They experienced some customers not staying for a long time, or leaving the venue, when they were warned and the smoke-free regulations were enforced. Moreover, some customers even insisted on smoking after being warned:

*‘We experience some reactions like, “I will smoke indoors, who will be angry with me?”.’* (ID 43, Restaurant owner, Ankara)

With smokers leaving the venue and non-smokers complaining about exposure to SHS, the staff admitted to being in a difficult position as they either lost customers or condoned the non-compliance:

*‘We are having issues with smokers among the customers, of course. He/she is sitting next to a closed window, there is a family at the next table, when we suggest changing his/her table they stand up and leave.’* (ID 54, Restaurant owner, Istanbul)

Furthermore, one participant highlighted that some customers feel justified in requesting to smoke in prohibited areas as they had been allowed to smoke in other venues/places that do not enforce the policy; this discourages some staff and venue owners to continue to implement the smoke-free policy:

*‘You try to explain this, and they say no. They (the customers) yell in front of all the (other) customers saying, “Hey man, then I go and sit at the place across. They (other venues) will allow this”.’* (ID 14, Restaurant owner, Istanbul)

*Economic concerns*

The challenges in confronting customers on an individual basis have left many venue owners/managers concerned about losing customers. One participant stated:

*‘The customer does not prefer us.’* (ID 27, Restaurant staff, Istanbul)

and another expressed concerns about the costs of providing a comfortable open space for smoking customers:

*‘Customers do not want to smoke in the cold. We literally have to warm the street, which has a cost.’* (ID 38, Restaurant owner, Ankara)

all these accumulate to larger concerns about maintaining their businesses.

*Physical properties of the venue*

Several respondents brought up the definition of enclosed areas in the smoke-free policy as a barrier in their efforts to comply with the regulations. They elaborated that the policy prohibits smoking in areas with automatic or manual awning systems or windows for ceilings and side walls, even if they can stay open during the day:

*‘The height of the wall is 60 cm to the windowsill [a glass wall]; it is totally an open space but still we are not compatible. We told them but they did not accept. They don’t want windows. They say it must be a garden. And very few places in Istanbul can accommodate this.’* (ID 14, Restaurant owner, Istanbul)

They added that the physical properties of their venue could not meet these standards and that having an ‘open space’ as described in the regulations was not possible for them. The current description of ‘open space’ is: ‘places without fixed or movable ceilings or roofs (such as open-air spaces), with at least three side surfaces permanently or temporarily open’. This type of description allows some, usually large, rooftop or garden access venues to have a ‘smoking open space’ while making such spaces very unlikely for small businesses. The respondents noted that if the regulations were equitably and forcefully enforced, worries about competitiveness would be greatly reduced.

*Insufficient enforcement*

All these concerns are tied to the main barrier that has been brought up by all our respondents: the issue of insufficient enforcement. The consensus among our participants was that the enforcement of the smoke-free policy was not being done or not being performed uniformly and effectively:

*‘The rules do not apply to everyone equally.’* (ID 48, Restaurant staff, Ankara)

*‘I think it works to the extent that the controls are done. It can be ignored if compulsory controls are not done.’* (ID 53, Restaurant staff, Istanbul)

In addition, the perception that violations have limited consequences due to insufficient enforcement causes some venue owners not to self-enforce the smoking ban as they witness unfair competition:

*‘I was shocked when I saw people smoke there [A shopping mall]. When very big brands that we can’t even compete with don’t do this, it would be complete nonsense that we do.’* (ID 14, Restaurant owner, Istanbul)

### COVID-19 pandemic and changes in compliance and implementation

All the above-mentioned issues were present before the COVID-19 pandemic occurred in the country. During the pandemic, all venues we interviewed were mandated to close for weeks and only re-open for take-away or delivery services for the following three months. This meant that small traditional tea and coffee shops that did not have deliverable menus had to remain closed, exacerbating economic difficulties.

We asked venue owners and employees how the pandemic changed their compliance practices. Several respondents talked about increased compliance among both venue staff and customers at the beginning of the pandemic. During the first few months of the pandemic, customers favored non-smoking places, were more reluctant to sit next to a smoker, and were more persistent in obeying the restrictions:

*‘People don’t come and sit where there’s someone smoking at the next table, or don’t go to crowded places here and there where people can smoke, they change their ways. They look from the door and don’t get in if someone is smoking inside.’* (ID 21, Restaurant staff, Istanbul)

In terms of implementation, while the majority stated that there were no changes, some participants noted an increased awareness and cooperation of customers. They gave examples of customers smoking less, not insisting on smoking, and responding positively when asked not to smoke in the prohibited area:

*‘Well, it made it easy for the smoking bans. At least when we said it to the customer, they were already afraid to take off their masks. Normally when they smoke cigarettes or water pipe, we have an excuse to warn them because of the pandemic, we say it’s forbidden and they fine us if we allow so they put the cigarette out.’* (ID 28, Restaurant staff, Istanbul)

On the other hand, some respondents highlighted the negative changes by describing the emotional and financial effects of the pandemic, due to restrictions for a long time, as barriers to implementation. They mentioned that customers were depressed and more prone to smoke while venues had less seating capacity – due to social distancing regulation – and were desperate for customers. Therefore, venues become more lenient with compliance:

*‘Some customers’ persistence is increased. Everybody is relaxed. Once they insist very much, we allow smoking one cigarette if they seem to leave the place.’* (ID 46, Owner of venue with waterpipe service, Istanbul)

### Recommendations for better implementation

Venue owners and staff were asked for their recommendations about what could be done by the government and the local municipality to improve the smoke-free policy implementation, as well as what they would do if they had the power to change things. Respondents’ responses could be categorized into two themes: the smoke-free policy content, and enforcement.

Türkiye’s smoke-free policy prohibits smoking in all indoor public places, including bars, restaurants, cafes, and shopping malls. This includes not only enclosed spaces but also semi-enclosed areas, such as covered terraces or balconies. The comprehensive scope of the prohibited areas is perceived to be causing difficulties in complying with the smoking ban. As a result, many suggested softening the indoor smoking ban and changing the law’s definition of enclosed areas:

*‘I think the government should leave people alone in this situation. Because people really got into financial difficulties within a year and a half, within two years. There are those (MoH regulators and local police officers) who act flexibly, there are also those who fine directly and go. If I were them, visiting places, I wouldn’t be so strict.’* (ID 50, Restaurant staff, Istanbul)

Some suggested ‘smoking areas’ or filtered air ventilation to allow smoking indoors:

*‘There can be signs on the door such as “smoking is allowed here” so that customers come accordingly. I mean like a smoking area. And I would like them to accept sliding awning. I think it would be enough if it is ventilated by 70%.’* (ID 48, Restaurant owner, Ankara)

There were also several suggestions for changing fines and regulation visit routines to be more deterrent and effective:

*‘Generally, like I said I would raise the fines a bit more because I think it is very little money.’* (ID 22, Café staff, Istanbul)

*‘The municipal police can do more frequent checks, outside and inside. They mostly work on complaints.’* (ID 21, Restaurant owner, Istanbul)

## DISCUSSION

This study explored the barriers to the implementation of Türkiye’s smoke-free policy from the perspective of hospitality venues. Findings suggest that the positive attitude of venue owners and staff toward the smoke-free policy serves as a facilitator. This is consistent with existing literature, which has shown that public awareness and support for smoke-free legislation contributed to effective smoke-free policy implementation^13^, and increased support is associated with knowledge of the harmful effects of SHS^14,15^ and the requirements of the smoke-free legislation^16,17^. Strengthening public knowledge about the adverse effects of SHS and the health benefits of the smoke-free policy can help hospitality venue owners and staff to better enforce the smoking ban.

The present study sheds light on four major barriers: fear of financial impact, customers’ negative attitudes, difficulties in meeting physical requirements, and insufficient enforcement. These barriers align with the findings of the Byron et al.^18^ review of smoke-free policy implementation in low- and middle-income countries, which identified insufficient capacity and financial support, poor enforcement, lack of implementation planning, limited public awareness as common barriers to implementing a smoke-free policy. So, while Türkiye is not alone in facing these difficulties, economic struggles brought on by the COVID-19 pandemic have indeed added a new layer of complexity to enforcing smoke-free policies in Türkiye and other countries.

Our findings also suggest the participants think that with such high levels of smoking in society, it is challenging to implement the smoke-free policy, which they believe will inevitably be violated. This finding aligns with a study conducted in Türkiye aimed at exploring café owners’ attitudes toward the smoke-free policy. The respondents mentioned that most of their customers were smokers and came to the café to smoke next to their beverages^19^. Likewise, studies^17,20,21^ revealed that pro-smoking norms (positive or accepting attitude towards smoking, including social acceptance and cultural traditions) and customer preferences could challenge the smoke-free policy implementation. In light of these findings, one main issue with the implementation of the Turkish smoke-free law is that it is yet to be fully accepted and internalized by the public. If not warned, people continue to smoke indoors. Thus, constant surveillance is necessary even 14 years after the passing of the law.

One of the important barriers found in our study was customers’ negative attitudes. Customers appeared to play a vital role in the fear of revenue loss in our study by preferring venues where they could smoke or leaving the venue when asked not to smoke in restricted areas. Financial concerns of venue owners have also been found to be a barrier in previous studies in Türkiye^19^, the United States^22^, China^23^, and Uganda^24^. However, a meta-analysis^25^ shows no economic impact in the long-term on employment or sales associated with smoking bans. The venue owner’s anxiety about revenue loss needs to be addressed by public health groups, and this issue needs to be openly discussed to alleviate these concerns. Research focusing on the economic impact of smoke-free policies on venues’ revenue in Türkiye could be undertaken. Moreover, public health groups can provide support by sharing success stories from other countries. Campaigns can highlight the positive impact on employee health, customer satisfaction, and overall business reputation, emphasizing the economic advantages of a smoke-free policy, such as increased customer loyalty and a healthier work environment.

Another significant barrier we found was insufficient enforcement, which led the venue owner/employee to think that implementing the policy was futile. They also resent the unfair competition with venues that do not enforce the policy. Furthermore, insufficient enforcement appeared to lead customers to consider breaking the rules as mundane/ordinary, and they become more likely to be argumentative when the rules are enforced. These results align with the challenges that Byron et al.^18^ also stated: poor enforcement, and lack of implementation planning. To address these issues, the government can take actions such as allocating sufficient resources to enforcement agencies, including personnel, equipment, and technology, to monitor and enforce smoke-free policies effectively. This may involve increasing the number of enforcement officers and providing them with the necessary tools to carry out their duties.

Participants reported the effect of COVID-19 on compliance with the implementation of the smoke-free policy to have varied. During the pandemic, hospitality venues in Türkiye closed temporarily or were limited to home deliveries. In the beginning, there were positive changes, with an initial increase in compliance among customers and staff. However, as the restrictions continued, negative changes emerged due to the emotional and financial strain. The interviews were conducted during the early stages of a gradual normalization process after a strict 6-month lockdown, and even then, a negative impact was observed. The economic struggles in Türkiye worsened, leading to rapid inflation and an increase in the cost of living. Stressed, customers were more inclined to smoke, and venue owners became more lenient in enforcing the policy. Respondents highlighted that these challenges resulted in decreased attention to smoke-free policy regulations among venue owners, staff, and customers. Currently, there is a scarcity of studies examining the effect of COVID-19 on compliance with smoke-free policies in hospitality venues. However, a study by Tian and Bakker^26^ investigating the influence of social media on promoting smoke-free policies in the catering industry during the pandemic in Beijing, underscored the effectiveness of social media campaigns in raising awareness, enforcing regulations, and monitoring violations. In the future, Türkiye could leverage the power of social media platforms as a potent tool to drive public health initiatives and establish a smoke-free environment.

Recommendations to reduce barriers to the implementation of smoke-free policies can include capacity-building programs for hospitality venue owners and staff to increase awareness about the benefits of comprehensive smoke-free policies for business, including improved air quality, reduced waste, and enhanced customer satisfaction. Social marketing campaigns targeting various parts of the population, in terms of age, education level, and other relevant segments, should highlight the importance of a 100% smoke-free environment for everyone, including a reduction in risk of heart disease, stroke, cancer and respiratory problems. Enforcement should be enhanced and consistent. The frequency of inspections should be increased by establishing a regular schedule of inspections for hospitality venues, with higher frequency for venues that have a history of non-compliance. Stricter sanctions should be implemented, including a graduated system of penalties for non-compliance, with increasing fines for repeat offenders. Addressing the perception among venue owners that they can avoid fines requires establishing a transparent inspection and fining process. Additionally, future studies focusing on the perspectives of enforcement officers, tobacco control committee members, and tobacco control NGO members regarding the facilitators and challenges of implementing smoke-free policies in Türkiye, could provide valuable insights and opportunities for improvement and collaboration.

### Limitations

There are some limitations in this study. Although ensuring diversity among venue types was considered the strength of this research, due to COVID-19 restrictions, bars, nightclubs, waterpipe and traditional tea houses were not fully operational at the time of the study. Thus, only a few waterpipe and traditional coffee houses were included, and no bars or nightclubs were visited for the study. Additionally, the study did not consider the smoking status of the participants. While the focus was on venue-level implementation of measures, smoking status could potentially influence individual interpretations and experiences. This aspect should be considered in future research to gain a more comprehensive understanding of the impact of smoking on the implementation of the policy. Furthermore, the study’s qualitative design may limit the generalizability of the findings to all types of venues in Türkiye. However, the qualitative design allows us to gain a deep understanding of the real-world experiences of participants and to uncover insights that may not be apparent from quantitative methods.

## CONCLUSIONS

This study explored how venue owners and staff perceive the enforcement of this law in the two largest cities of the country, 14 years after the initiation of the smoke-free policy. Our findings illustrate the difficulties in putting smoke-free policy measures into practice and keeping them in place over the years. It also highlights additional complications introduced by the COVID-19 pandemic. Recommendations to improve compliance at multiple levels were made.

## Data Availability

The data supporting this research are available from the authors on reasonable request.
