# Supplementary material for: Understanding the dynamics of compliance to smoke-free policy regulations: Exploring the perspectives of venue owners and staff in Türkiye
Source: Tob Induc Dis. 2024 Jan 17;22:10.18332/tid/176226. doi: 10.18332/tid/176226 (PMC10792618; doi:10.18332/tid/176226)
Supplement: Supplementary file 1 [file TID-22-11-s1.pdf]

Supplementary Material

Supplementary Material-1. The interview guide

**Open ended survey**
What do you know about Turkey's smoke-free regulations?
To what extent do you think it works?
We hear people say that it is not good for business. But in your opinion, is there anything that is good about it?
How would you rate your venue's compliance level with the smoke-free regulations?
Would you say that cigarette and/or waterpipe smoke is totally eliminated from all the enclosed spaces, at all working hours in your work environment?
How did this level of compliance change with COVID-19
What were the problems you face when implementing this smoke-free airspace regulation?
Have these problems change with COVID-19? New problems?
Has it become easier in any way after COVID-19?
What can be done by the government to make the implementation easier for you?
In your opinion, is there anything that your local municipality can do to make your implementation easier?
Have you had any visitors from the tobacco sellers (BAT or Phill Morris) to add stands, to extend or renew existing stands.
If you had the power to change things what would you do about this regulation?
If you had to decrease the smoke and create good air quality in work places like yours, what would you do?

© **2024 Baltacı E. et al.**
